# Temporal trends in the initiation of glucose-lowering medications after a first-time myocardial infarction - a nationwide study between 1997 and 2006

**DOI:** 10.1186/1475-2840-10-5

**Published:** 2011-01-19

**Authors:** Mette L Norgaard, Charlotte Andersson, Peter Riis Hansen, Søren S Andersen, Allan Vaag, Tina K Schramm, Fredrik Folke, Lars Køber, Christian Torp-Pedersen, Gunnar H Gislason

**Affiliations:** 1 Department of Cardiology, University Hospital of Copenhagen, Gentofte, Denmark; 2 Steno Diabetes Center, Gentofte, Denmark; 3 The Heart Centre, Rigshospitalet, University of Copenhagen, Denmark; 4 Department of internal medicine, University Hospital of Copenhagen, Herlev, Denmark; 5 Faculty of Health Sciences, University of Copenhagen, Denmark

## Abstract

**Background:**

Type 2 diabetes is a well-established risk factor for cardiovascular disease and is common among patients with acute myocardial infarction (MI). The extent to which patients with first-time MI develop diabetes requiring glucose-lowering medications (GLM) is largely unknown. The aim of the study was to investigate temporal trends in the initiation of GLM among patients discharged after first-time MI.

**Methods:**

All Danish residents aged ≥ 30 years without prior diabetes hospitalized with first-time MI between 1997 and 2006 were identified by individual-level-linkage of nationwide registers. Initiation of GLM during follow-up was assessed by claimed prescriptions from pharmacies. Temporal trends in initiation of GLM were assessed by incidence rate calculations in the MI population as in the general population. Multivariable Cox proportional-hazard models were used to investigate the likelihood of initiating GLM within a year post-MI.

**Results:**

The population comprised 66,788 patients. Among these patients 3962 patients initiated GLM, of whom 1567 started within one year post-MI. An increase in incidence rates of GLM initiation in the MI population from 19.6 per 1000 person years in 1997 to approximately 27.6 in 2001 was demonstrated. After 2001 the incidence rates stabilized. A similar trend was observed in the general population where the incidence rates increased from 2.8 in 1997 to 4.0 in 2004 and then stabilized.

**Conclusion:**

Our study demonstrated an increase in incidence rates of GLM initiation within the first year post- MI. A similar trend was observed in the general population suggesting that the increase in GLM among MI patients was primarily the effect of a general increased awareness of diabetes. From a public heath perspective, this study underscores a continuous need for diagnostic and therapeutic improvement in the care of MI patients that develop diabetes.

## Background

Type 2 diabetes is a well-established risk factor for cardiovascular disease and is common among patients with acute myocardial infarction (MI), where the prevalence is as high as 20%[1]. Previous studies have shown that abnormal glucose metabolism is more common than normal glucose tolerance among patients with MI and that abnormal glucose tolerance is an important predictor of impaired long-term outcome in these patients[2-4]. Thus, among patients with MI without known diabetes up to 65% exhibit abnormal glucose regulation when challenged with an oral glucose tolerance test (OGTT) and 25% of these have values diagnostic for diabetes and 40% have impaired glucose tolerance[5]. Focus on the adverse prognosis carried by diabetes in post-MI patients has increased over the years, causing new guidelines to be developed in 2007. These recommend that patients without diabetes admitted with MI are investigated with an OGTT within 3 months post MI[5,6]. However, information regarding the actual number of patients receiving glucose-lowering medications (GLM) after first-time MI on a population basis is lacking. We therefore conducted the present nationwide register-based study to investigate the temporal trends in the initiation of GLM following hospitalization for first-time MI between 1997 and 2006.

## Methods

### Population and Data Sources

All residents in Denmark have a unique and permanent civil registration number that enables individual-level-linkage between nationwide registers. The study population was identified employing the Danish Civil Registration system. All medications dispensed from pharmacies were obtained from the National Prescription Register (the Danish Register of Medicinal Product Statistics), which holds information on all prescriptions dispensed from Danish pharmacies since 1995. The prescription register keeps information on date of dispensing, strength and quantity. All drugs are coded according to the *Anatomical Therapeutic Chemical *(ATC) classification system. Information on hospital admissions was obtained from the Danish National Patient Register, which keeps records on all hospital admissions in Denmark since 1978 [7]. Each hospitalization is registered at discharge with diagnoses recorded using the *International Classification of Diseases *(ICD), until 1994 the 8^th ^revision (ICD-8) and from 1994 the 10^th ^revision (ICD-10). All deaths were identified from the Central Population Register where deaths are recorded within 2 weeks.

The study population comprised all patients aged 30 years or older at the start of the study period and admitted to the hospital between January 1, 1997 and 31, December, 2006 with a primary or secondary diagnosis of first-time MI (ICD-10 code I21, I22). First-time admission for MI implied that the National Patient Register had not registered any prior admission for MI in the previous 19 years. To fully ensure that data available from the National Prescription Register were complete inclusion of patients were selected from the year 1997. The diagnosis of MI in the National Patient Register has proved to be valid with a sensitivity of 91% and a positive predictive value of 93% [8]. Diabetes was defined according to prescription claims for GLM. Thus, patients who had claimed any prescriptions for GLM prior to MI hospitalization were not included and patients that developed diabetes requiring GLM were identified as individuals initiating GLM (oral or insulin; ATC code A10) after first-time MI during the study period, i.e. between January 1, 1997 and December 31, 2006. Patients receiving GLM prior to January 1, 1997 were excluded.

The study population was divided into five two-year periods in order to study the temporal trends in initiation of GLM.

The general population comprised all individuals at least 30 years of age and alive on January 1, 1997. The population was updated on a yearly basis allowing entrance of new subjects at least 30 years of age per January 1, the following year. Patients with a previous MI as well as patients with incident MI were excluded from the general population as were patients treated with GLM prior to January 1, 1997.

### Comorbidity

Comorbidity was assessed using the Ontario Myocardial Infarction Mortality Prediction Rules for ICD-10 codes based on diagnoses from the index hospitalization with MI and up to one year prior to discharge from index hospitalization (diabetes diagnoses excluded)[9].

### Glucose-lowering medication

For determination of first initiated GLM, the following agents (ATC-codes) were identified: insulin (*A10A*), metformin (*A10BA02*), sulfonylureas (*A10BB*), glitazones (*A10BG,; *acarbose (*A10F01*), repaglinide (*A10BX0) *and metformin+repaglinide (*A10BD03*). Since only 47 patients were treated with glitazones, acarbose, repaglinide and metformin + repaglinide as their first GLM, we combined these treatment groups in one group denoted '*other GLM'*.

### Statistical analyses

Continuous variables are presented as mean with standard deviation (SD). Categorical data are presented as percentages. The demographic characteristics are presented for the five study periods outlined above.

Incidence rates (per 1000 person-years) in initiation of GLM within a year post-MI were calculated leaving out year 2006 due to incomplete follow-up time. To investigate whether the temporal trends in treatment were an effect of increased focus of diabetes in MI patients, or whether similar trends were seen in the general population, incidence rates in initiation of GLM was also calculated in the general population.

Multivariable Cox proportional-hazard models (adjusted by age, gender calendar year) were used to investigate the likelihood of initiating GLM within a year post-MI according to the different treatment years. In this analysis the study period was divided into five two-year periods using year 1997-98 as the reference group.

Temporal trends in initiation of different pharmacological types of first initiated GLM was done in percentages also with the study period divided into five two year periods. Test for temporal trends in pharmacological types of first initiated GLM initiated were done by Cochran-Armitage trend test.

All statistical calculations were performed with SAS statistical software package, version 9.1 (SAS Institute Inc, Cary, NC) and STATA version 11.0 (StataCorp, College Station, Texas).

### Ethics

The Danish Data Protection Agency approved the study (No. 2007-41-1667). Retrospective register based studies in which individuals cannot be identified do not need ethical approval in Denmark.

## Results

### Population

In total 89,448 patients ≥ 30 years of age were diagnosed with first-time MI during the study period. Of these, 77,147 had never used GLM prior to study start. In total 66,788 were alive at discharge and therefore formed the present study population.

A total of 3962 patients initiated GLM treatment during the study period. Within the first year post discharge from first-time MI 1567 patients initiated GLM. A total of 1452 patients were included in the incidence rate analysis since the 115 patients initiating GLM in 2006 were excluded, due to incomplete follow-up time. As shown in Table 1, the study population comprised an excess of males and the mean age was 68-69 years in all five study cohorts. Information on comorbidity is also listed in Table 1.

**Table 1:** Demographic characteristics at the time of first-time myocardial infarction according to year of occurrence.

	1997-1998	1999-2000	2001-2002	2003-2004	2005-2006
Patients with first-time MI^a^	11 968	12 383	14 734	14 650	13 053
Patients initiating GLM^b ^within a year post-MI	227	266	389	389	296
Men (%)	64.0	62.4	61.6	62.1	62.4
Mean age (SD)	67.8 (12.9)	68.5 (13.1)	69.3 (13.1)	69.4 (13.2)	69.4 (13.2)
Comorbidity^c^					
Congestive heart failure (%)	9.3	12.1	12.9	11.4	10.3
Cardiac arrhythmias (%)	7.3	9.4	10.9	11.5	11.1
PVD^d ^(%)	1.9	2.3	2.5	2.3	2.3
CVD^e ^(%)	3.4	4.5	4.9	5.1	4.8
Renal disease (%)	0.8	1.7	2.1	2.3	2.3
Cancer (%)	2.0	2.4	2.9	2.9	2.9
Shock (%)	0.6	0.7	1.2	1.3	1.3
COLD^f ^(%)	4.4	6.0	7.4	7.0	6.4
Peptic ulcer	1.5	1.5	2.0	1.6	1.6

^a^MI: myocardial infarction; ^b ^GLM: glucose-lowering medication; ^c^comorbidity up till one year prior to discharge from index hospitalization; ^d^PVD: peripheral vascular disease; ^e ^CVD: cerebrovascular disease; ^f ^COLD: chronic obstructive lung disease.

### Temporal trends in the incidence of GLM initiation

**Figure 1:**
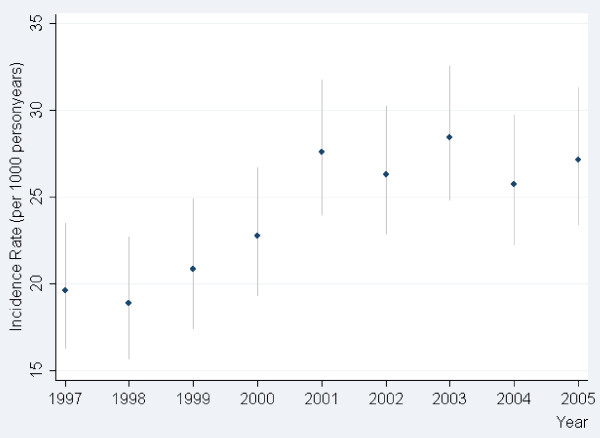
**Incidence rate per 1000 person years in initiation of glucose lowering medication (GLM) within a year following for first-time myocardial infarction**.

In the year 1997 the incidence rate of GLM initiation within the first year after discharge for first-time MI was approximately 19.6 per 1000 person years. This incidence rate increased until year 2001 (incidence rate 27.6). From 2001 and onwards the incidence rate stabilized, Figure 1.

The probability of initiation of GLM within the study period increased to a maximum in 2003-2004, HR corresponding to 1.42 (95% CI 1.20-1.67; p-value < 0.0001), when using year of 1997-1998 as a reference, Table 2. This corresponds to the highest incidence rate of GLM initiation in 2003 of 28.4 per 1000 person years.

**Table 2:** Adjusted Cox proportional hazard model: Probability of initiating Glucose-lowering medications within a year post-myocardial infarction adjusted by age, gender calendar year and stratified by year group.

Variable	Hazard Ratio	95% CI	P
1997-1998	1.00		
1999-2000	1.13	0.95-1.35	0.17
2001-2002	1.40	1.19-1.65	<0.0001
2003-2004	1.42	1.20-1.67	<0.0001
2005-2006	1.37	1.15-1.63	0.0004

**Figure 2:**
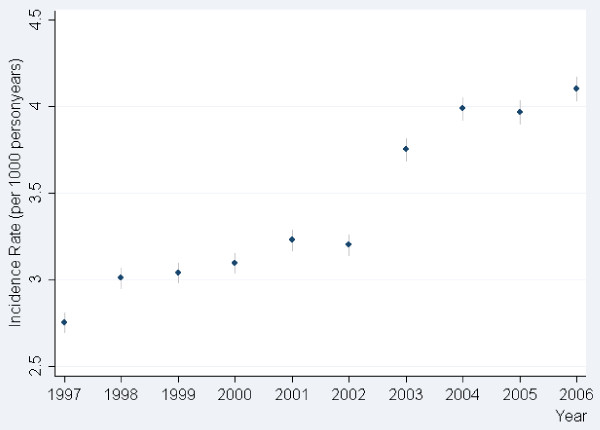
**Incidence rate per 1000 person years in initiation of glucose lowering medication (GLM) in the general population**.

A similar trend in increased incidence rates was observed in the general population. The incidence rate increased from 2.8 to 4.0 per 1000 person years in 2004. Following year 2004 the incidence rate stabilized, Figure 2.

### Temporal trends in type of first initiated GLM

In the entire population initiating GLM after first-time MI, the two predominantly prescribed GLMs were sulfonylureas and metformin, Table 3. During the study period the use of metformin as first GLM increased significantly from 30.5% in 1997-1998 to 40.0% in 2005-2006 (p for trend < 0.0001). Conversely, the use of sulfonylureas decreased from 59.0% in 1997-1998 to 45.5% in 2005-2006 (p for trend < 0.0001). The use of insulin as first-GLM also increased from 9.1% in 1997-1998 to 14.3% in 2005-2006 (p for trend < 0.002).

**Table 3:** Distribution of first prescription claimed for glucose-lowering medications according to year of first-time myocardial infarction.

	1997-1998	1999-2000	2001-2002	2003-2004	2005-2006	p for trend.
Metformin (%)	30.47	33.83	36.62	38.03	39.94	<0.0001
Insulin (%)	9.07	9.59	12.70	13.25	14.29	0.002
Sulfonylurea (%)	59.07	55.43	48.90	48.01	45.48	<0.0001
Other GLM(%)^a^	1.38	1.16	1.78	0.71	0.29	0.15

^a ^GLM: glucose-lowering medications: Other GLM: glitazones, acarbose, repaglinide, and repaglinide + metformin.

## Discussion

Our study demonstrated an increase in the incidence rate of GLM initiation within the first year after first-time MI from 19.6 per 1000 person years in 1997 to 27.6 per 1000 person-years in 2001. Following year 2001 the incidence rates stabilized. The increased probability of initiating GLM after a first-time MI was confirmed in adjusted Cox-analyses. A similar trend in the increased incident rate in GLM initiation was demonstrated in the general population from 2.8 per 1000 person years in 1997 to 4.0 per 1000 person years in 2004. Corresponding to the MI population a stabilisation in incidence rate was observed in the general population after 2004. The international diagnostic criteria for diabetes was changed from a fasting blood glucose value of 7.0 mM to 6.1 mM in 1997, marking the initiation of this nation-wide observational study, why we cannot exclude the possibility that an unknown proportion of the increased incidence of diabetes during the period from 1997 until 2001 was in fact due to the subsequent implementation in clinical practice of these novel diagnostic criteria in Denmark. However, it should be noted that the criteria on diabetes/pre-diabetes was not changed in Denmark during the study period,[10], opposite to what happened in the US in 2003, where the cut-off point of impaired fasting glucose was further lowered to 5.6 mmol/L by the American Diabetes Association (ADA)[11]. Nevertheless, the theoretical possibility that the increased general use of GLM during the period from 1997 until 2001 to some (unknown) extent may be due to the implementation more stringent diagnostic criteria for diabetes, in fact support and substantiate the main conclusion of the current work being that diabetes remain disproportionately under diagnosed and under treated among the high risk proportion of patients developing diabetes after a MI.

Previously, studies in MI patients have documented that abnormal glucose metabolism before discharge is more frequent than normal glucose metabolism [2,3,12]. Indeed, *the Euro Heart Survey on Diabetes and the Heart *with data from 25 countries disclosed unrecognized diabetes in 22% of patients with acute coronary syndrome according to results of the OGTT [3,13]. In addition, Norhammar et al. conducted a small-scale prospective study of 181 consecutively admitted MI patients without known diabetes, where an OGTT was performed at discharge and again 3 months later. According to the OGTT, 35% and 40% had impaired glucose tolerance at discharge and after 3 months, respectively and 31% and 25% had previously undiagnosed diabetes [11]. Similar results were also found by Egstrup et al [2,14].

These results indicate that a large proportion of post-MI patients have undiagnosed diabetes or develop diabetes during admission or within a short time-span after discharge. We therefore anticipated that a much larger proportion of patients would have initiated GLM shortly after discharge for MI, than observed in our current study. There are numerous possible explanations for this discrepancy. First, diabetes in many patients was probably left undiagnosed, because the majority of patients presented with normal fasting glucose values and therefore in accord with contemporary guidelines, did not undergo an OGTT, although many would here have impaired glucose tolerance or overt diabetes [2]. Importantly, however, because our study was conducted prior to publication of prevailing international guidelines, these numbers should not be interpreted as representing a predominant negligence from clinicians. Second, guidelines regarding management of newly detected diabetes at the time of the study generally recommended lifestyle intervention and dietary treatment as first-line treatment. Therefore it is conceivable that a substantial number of patients not initiating GLM were in fact being managed by non pharmacological intervention. However, since diabetes is well-established as an important independent long-term prognostic factor after MI that predicts increased mortality even 17 years after MI,[15] our study underscores the importance of an aggressive diagnostic and therapeutic approach in diabetes patients with MI. It is highly unlikely that lifestyle interventions were sufficient for all diabetes patients not initiating GLM to have reached target blood glucose values, and our results indicate that a considerable number of post-MI patients do not receive GLM as appropriate. This is, however, only an assumption as we e.g. do not have any measurements of blood glucose values during and after hospitalization.

In agreement with the results cited above, treatment guidelines have recommended that all patients hospitalized with MI have a fasting glucose measurement during hospitalization, and are subjected to an OGTT three months after discharge [5,6]. Furthermore, management of patients with diabetes with lifestyle interventions (including dietary treatment) alone is no longer considered to be first- line treatment. In addition to such interventions, patients with newly diagnosed diabetes should initiate treatment with metformin unless contraindicated [16]. Therefore, it would be of great interest to repeat the current study in the future, investigating the impact of the change of treatment guidelines in this real life population-scale setting.

During the 10 year study period a considerable increase in initiation of GLM in the general population was apparent. The incidence of diabetes in our study was in consistency with a previous study employing the Danish National Diabetes Register, where Carstensen et al. demonstrated an increase in the incidence of diabetes [17]. Whether the increase in incidence of diabetes in Denmark represents a true epidemiologic increment in diabetes rates is questionable, and other possible explanations include 1) increased diagnostic intensity due to an augmentation of the awareness of diabetes in the general population, and 2) a large undiagnosed population of patients with diabetes early in the study period was being diagnosed during the rest of the study period, and 3) a decrease in mortality rates in the general population leading to increased numbers of subjects at risk of diabetes. The extent to which these individual factors each contribute to the increase in incidence of diabetes is speculative. A very important discrepancy in our study compared with the study by Carstensen et al. was that we only documented an increase in incidence of patients with diabetes requiring GLM. Supplementary to the explanations outlined above, we speculate that an increased awareness during the 10 year study period of the importance of reaching normoglycaemia in diabetes patients with use of GLM may also contribute to our finding of increased incident diabetes.

We would have anticipated a steeper increase in incidence rate in the post-MI population compared to the general population. Indeed the incidence rates are much higher in the MI population than in the general population, which is not surprising, since MI patients are at much higher risk of developing diabetes and since diabetes is a risk factor for MI. If the higher incidence rates in the MI population compared to the general population were solely to be explained by a true increased awareness of diabetes in the MI population and not in the general population as a whole, a steeper, linear or even exponential increment of incident diabetes in the MI population would be expected. Since we did not find such steep increase, it is most likely that the increased initiation of GLM in the MI population was, in fact, primarily the effect of a general increased awareness of diabetes in the population as a whole.

Results from randomized controlled trials have conclusively demonstrated that the risk of microvascular complications can be reduced by intensive glycaemic control in patients with diabetes [18-22]. Although the effect of intensive glycaemic control on macrovascular outcomes remains a matter of intense debate,[23-25] the American Diabetes Association (ADA) currently stated that glycaemic control early in the course of type 2 diabetes may provide CVD benefits and that glycaemic control may play a greater role for CVD prevention in patients with a shorter duration of diabetes before macrovascular disease is established [26].

It is known that patients with diabetes and cardiovascular disease tend to have poorer glycaemic control than those with diabetes without cardiovascular disease, indicating the need for increased attention to glycaemic control in patients with cardiovascular disease [27]. The current results shows that when it comes to early GLM initiation in those with newly detected diabetes, there remains a considerable room for improvement in modern clinical practice.

The change in the pattern of type of GLM used in MI patients during the period is likely to reflect the results of the UKPDS as well as the Diabetes Mellitus, Insulin Glucose Infusion in Acute Myocardial Infarction Study 1 signaling increased survival among users of metformin in general as well as the value of early insulin treatment after a recent MI [21,28]. This has shown to be valid also for patients undergoing percutaneous intervention (PCI), in whom the use of metformin or thiazolidinediones seem to attenuate the re-stenosis formation [13]. Opposite, the effect of sulfonylureas or insulin on outcomes in MI patients treated with and without PCI seems to be less beneficial [29,30]. The extent to which our findings of changes in clinical practice may change the long-time survival in Danish MI patients with diabetes remain to be determined.

### Strengths and limitations

This study is based on complete and nationwide data obtained from registers that previously have been validated and shown to be reliable. The data cover the entire population of Denmark independent of socioeconomic status, age or participation in specific health-insurance programs. Therefore, the risk of selection bias is reduced, and the study notably includes citizens both in and out of the labor market. The Danish health care system partially reimburses drug expenses, and all Danish pharmacies are required to register all dispensed drug prescriptions, ensuring complete registration. There are, however, important limitations that need to be addressed. We lack information about additional factors influencing the decision for initiation of GLM, and other unmeasured confounders. Furthermore, we were unable to identify type 1 and type 2 diabetes among the insulin users and we could not assess the duration of diabetes prior to initiation of GLM in our study, since the registers do not hold information on HBA1c, fasting blood glucose, and other clinical characteristics, e.g. body mass index (BMI).

The diagnostic criteria for MI changed in 1999 and more sensitive diagnostic markers were introduced (i.e. troponins) during the study period, which potentially may have influenced our results, although e.g. a previous study indicated no change in survival for MI patients [31].

## Conclusions and clinical implication

Our study demonstrated an increase in incidence rates of GLM initiation within the first year post- MI. Parallel, a similar trend was observed in the general population. These results indicate that the increased initiation of GLM in the MI population was primarily the effect of a general increased awareness of diabetes in the population as a whole. From a public heath perspective, this study underscores a continuous need for diagnostic and therapeutic improvement in the care of MI patients that develop diabetes, and such strategy is likely to provide long-term benefits.

## Abbreviations

ATC: Anatomical Therapeutic Chemical; GLM: glucose lowering medications; CVD: cardiovascular disease; DCCT-EDIC: Epidemiology of Diabetes Intervention and Complications trial; ICD: International Classification of Diseases; MI: myocardial infarction; OGTT: oral glucose tolerance test; UKPDS: the United Kingdom Prospective Diabetes Study;

## Competing interests

A. Vaag is employed by Steno Diabetes Center, which is owned by Novo Nordisk A/S. All other authors declare that they have no competing interests.

## Authors' contributions

All authors read and approved the final manuscript. *Study concept and design and acquisition of data: *MLN, CA, CTP and GG. *Analysis and interpretation of data: *MLN, CA, CTP, GG, SSA, TKS, FF AVAA, PEHA and LK. *Drafting of the manuscript: *MLN. *Critical revision of the manuscript for important intellectual content: *MLN, CA, CTP, GG, SSA, TKS, FF, AVAA, PEHA and LK. *Statistical analysis: MLN, CA, CTP and GG*. *Obtained Funding: *MLN, CTP and GG. *Administrative, technical, or material support: *MLN, CTP and GG. *Study supervision: *CTP and GG

Institution where work was performed:

Department of Cardiology, Gentofte Hospital, Gentofte, Denmark
